# Bioelectrocatalysis with a palladium membrane reactor

**DOI:** 10.1038/s41467-023-37257-7

**Published:** 2023-03-31

**Authors:** Aiko Kurimoto, Seyed A. Nasseri, Camden Hunt, Mike Rooney, David J. Dvorak, Natalie E. LeSage, Ryan P. Jansonius, Stephen G. Withers, Curtis P. Berlinguette

**Affiliations:** 1 Department of Chemistry, The University of British Columbia, 2036 Main Mall, Vancouver, BC V6T 1Z1 Canada; 2 Stewart Blusson Quantum Matter Institute, The University of British Columbia, 2355 East Mall, Vancouver, BC V6T 1Z4 Canada; 3 Department of Chemical and Biological Engineering, The University of British Columbia, 2360 East Mall, Vancouver, BC V6Y 1Z3 Canada; 4 Canadian Institute for Advanced Research (CIFAR), 661 University Avenue, Toronto, M5G 1M1 ON Canada

**Keywords:** Hydrogen energy, Electrocatalysis, Biocatalysis, Electrochemistry

## Abstract

Enzyme catalysis is used to generate approximately 50,000 tons of value-added chemical products per year. Nearly a quarter of this production requires a stoichiometric cofactor such as NAD^+^/NADH. Given that NADH is expensive, it would be beneficial to regenerate it in a way that does not interfere with the enzymatic reaction. Water electrolysis could provide the proton and electron equivalent necessary to electrocatalytically convert NAD^+^ to NADH. However, this form of electrocatalytic NADH regeneration is challenged by the formation of inactive NAD_2_ dimers, the use of high overpotentials or mediators, and the long-term electrochemical instability of the enzyme during electrolysis. Here, we show a means of overcoming these challenges by using a bioelectrocatalytic palladium membrane reactor for electrochemical NADH regeneration from NAD^+^. This achievement is possible because the membrane reactor regenerates NADH through reaction of hydride with NAD^+^ in a compartment separated from the electrolysis compartment by a hydrogen-permselective Pd membrane. This separation of the enzymatic and electrolytic processes bypasses radical-induced NAD^+^ degradation and enables the operator to optimize conditions for the enzymatic reaction independent of the water electrolysis. This architecture, which mechanistic studies reveal utilizes hydride sourced from water, provides an opportunity for enzyme catalysis to be driven by clean electricity where the major waste product is oxygen gas.

## Abstract (web summary)

Enzymatic catalysis requires cofactors such as NAD(P)H, whose regeneration is currently accomplished via secondary enzymes or electrolytic cell. Here, the authors report an electrochemical method of cofactors regeneration without supporting enzymes or mediators, nor formation of NAD_2_ dimers.

## Introduction

**Fig. 1:**
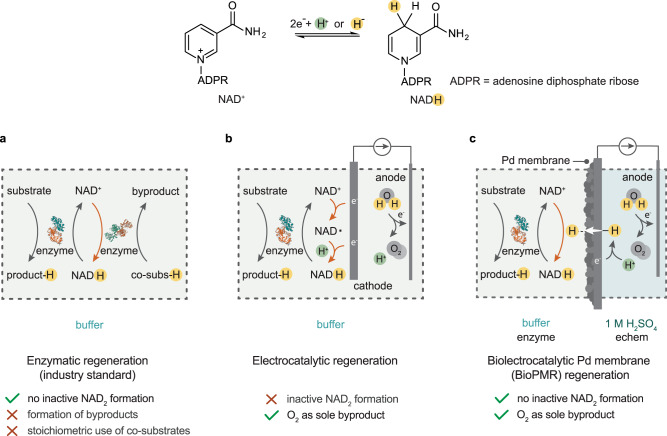
Schematic representation of a, enzymatic, b, electrocatalytic, and c, bioelectrocatalytic palladium membrane reactor (BioPMR) NADH regeneration. The BioPMR functions as follows: (i) proton reduction occurs on the Pd membrane to form H atoms; (ii) H atoms diffuse through the Pd membrane; and (iii) surface-bound H atoms react with NAD^+^ to form NADH which is subsequently used for enzyme catalysis. Orange arrows indicate the NADH regeneration step(s) in each method.

Enzyme catalysis enables challenging chemical reactions to be performed with a high activity and selectivity at ambient conditions^1–3^. The industrial use of enzymes for chemical production currently represents a 100 billion USD industry^4^. A major application of enzyme catalysis is enantioselective synthesis in the pharmaceutical sector, with two thirds of commercial chiral products involving enzyme catalysis^5^. Up to 25% of known classes of enzymes require costly stoichiometric cofactors such as nicotinamide adenine dinucleotide (NAD^+^) and its reduced form (NADH)^6–8^. These stoichiometric cofactors provide hydride as a reagent (Fig. 1) and must be continuously regenerated in situ for viable catalysis^9,10^. Cofactor regeneration in the industry is currently on performed with secondary enzymes (e.g. formate and glucose dehydrogenases) that catalyze NADH formation from NAD^+^ using stoichiometric co-substrates such as formate or glucose (Fig. 1b)^11,12^. These systems require the separation of water-soluble co-substrates and their byproducts, which results in high regeneration costs and limits their application to the synthesis of high-value products^13,14^. The development of alternative cofactor regeneration methods that do not require the separation of co-substrates and byproducts is a challenge for the design of low-cost enzymatic catalytic systems.

An alternative method for regenerating NADH is electrocatalytic regeneration. Protons and electrons sourced from water electrolysis can produce reduced cofactors (e.g., NAD^+^ + 2e^–^ +H^+^ → NADH) directly at a cathode surface, without the use of secondary enzymes or co-substrates (Fig. 1b)^15–21^. This process occurs in two steps: (i) A one-electron reduction of NAD^+^ to NAD• beginning at –0.6 V vs. standard hydrogen electrode (SHE); and (ii) a proton-coupled reduction of NAD• to NADH at –1.4 V vs. SHE (Fig. 1b)^22^. This two-step process ideally produces NADH at the cathode and O_2_ at the anode as the sole byproduct. However, NAD• formed during step (i) rapidly dimerizes to form inactive NAD_2_^22^. Dimerization can be mitigated by applying a high cathode voltage (–1.4 to –1.8 V vs. SHE) to accelerate step (ii)^23^. However, this high voltage introduces new complications in the form of heat and a strong electric field at the electrode surface, both of which can inactivate and/or denature enzymes^24–27^. A mediator can be added to prevent NAD^+^ dimerization and bypass the need for a high cathode voltage^28,29^, but this mediator reintroduces separation challenges similar to those found in enzymatic regeneration^10^, unless the mediator is immobilized on the electrode surface^30^. The development of electrocatalytic cofactor regeneration methods that completely circumvent NAD• formation would enable NADH regeneration to be performed without high cathode voltages or the use of a mediator, while producing O_2_ as the sole byproduct. A system that meets these criteria could potentially supplant the use of secondary enzymes for cofactor regeneration.

Here, we demonstrate that a bioelectrocatalytic palladium membrane reactor (BioPMR) represents such a system (Fig. 1c). The BioPMR consists of an electrochemical compartment and an enzymatic compartment, with a palladium foil separating both compartments. The palladium foil functions as: (i) the cathode of a 3-electrode electrochemical cell; (ii) a hydrogen-selective membrane; and (iii) a catalyst for cofactor regeneration. Electrolysis performed in the electrochemical compartment generates hydrogen at the palladium foil, which then permeates through the foil and reacts with NAD^+^ to regenerate NADH for consumption by the enzyme in the enzymatic compartment (Fig. 1c). We find that the NADH regeneration process in the BioPMR occurs through direct hydride (H^−^) transfer. This mechanism is distinctive from the 2-step electron-transfer processes characteristic of previously reported electrochemical cells where regeneration is driven by the electrochemical bias at the electrode. A benefit of NADH formation through hydride transfer is that NAD_2_ formation is bypassed even at low reductive potentials (–0.3 V vs. SHE). Moreover, the physical separation of the compartments enables conditions for the electrolytic (i.e., highly acidic or basic) and enzymatic (i.e., physiological pH) processes to be independently optimized. This separation also increases the durability of the system by isolating the enzyme from the electrochemical processes. We demonstrate these advantages herein by performing the following enzymatic processes at their optimal pH in the BioPMR: (i) aldehyde hydrogenation; (ii) ketone hydrogenation, and; (iii) reductive amination. The outcomes of this study show that this platform represents an approach for achieving low-waste, enzyme-catalyzed reactions.

## Results

**Fig. 2:**
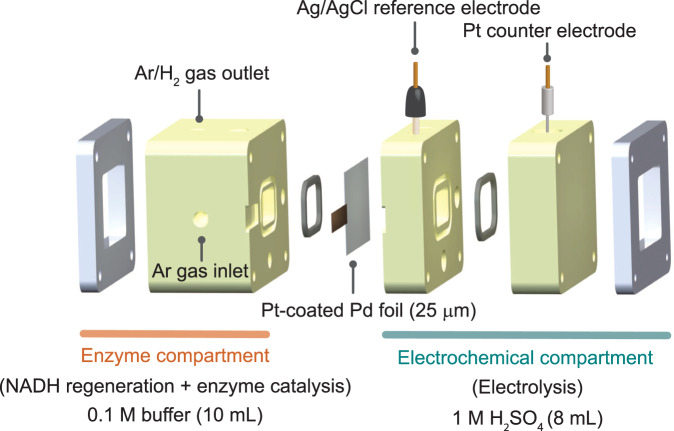
3D-printed reactor design of the BioPMR. The 2-compartment cell contains an electrochemical compartment filled with 1 M H_2_SO_4_ and an enzyme compartment filled with buffer solution. The electrochemical compartment contains a platinum anode, a Ag/AgCl reference electrode, and the palladium cathode/membrane. Catalyst (Pd black or Pt-coated) is coated on the side of the membrane exposed to the enzyme compartment.

The BioPMR cell (Fig. 2) consists of an electrochemical compartment and an enzyme compartment. The electrochemical compartment contains a 3-electrode cell with a Ag/AgCl reference electrode, a Pt mesh counter electrode (where water oxidation occurs), a 25-µm thick Pd foil working electrode (where proton reduction and hydrogen absorption occur), and electrolyte (8 mL of 1 M H_2_SO_4_)^31^. The Pd foil also functions as a hydrogen-selective membrane separating both compartments. The enzyme compartment (where the hydrogen that diffuses through the membrane reacts with NAD^+^) contains buffer solution (10 mL of 0.1 M phosphate or tris buffer), 1.5 mM NAD^+^, enzyme, and 1.5 mM substrate. The side of the palladium membrane facing the enzyme compartment is coated with electrodeposited Pd black to increase the active area^31^. The Pd black layer is further coated with sputtered Pt (10-nm thick). Details regarding the cell construction can be found in the SI. Details regarding the choice of enzyme and substrate are found below.

**Fig. 3:**
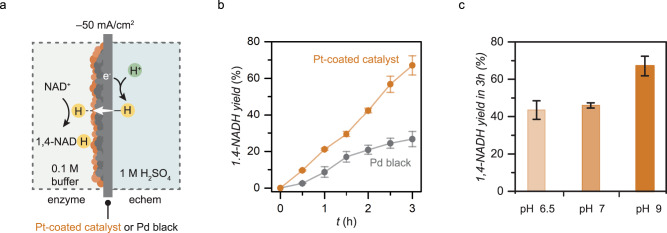
NADH generation performance in the ePMR. **a** Cell setup and idealized process for NADH generation. **b** Reaction profile for 1,4-NADH generation with Pd black catalyst and Pt-coated catalyst (Pt/Pd black) in tris buffer at pH 9. **c** 1,4-NADH yield in 3 h with Pt-coated catalyst as a function of buffer pH (phosphate buffer for pH 6.5, tris for pH 7–9). Error bars represent the standard deviation of triplicate measurements. The center point of each error bar represents the mean of the triplicate measurements.

We first demonstrated that the BioPMR enables the generation of NADH from NAD^+^ using water as a proton and electron source. A solution of 1.5 mM NAD^+^ and 0.1 M tris buffer (pH_measured_ = 9.0) was added to the enzyme compartment and 1 M H_2_SO_4_ was added to the electrochemical compartment (Fig. 3a). The cell was configured as described above (Fig. 2) and heated to physiological temperature (37  ^°^C) in an oven. Galvanostatic electrolysis was performed (*I*_applied_ = –50 mA cm^–2^) under Ar gas. We initially confirmed the formation of the biologically active NADH isomer (i.e. 1,4-NADH) in the enzyme compartment with a Pd black catalyst by UV–Vis absorption spectroscopy (*λ*_max_ = 340 nm, Supplementary Fig. 1) and a lactate dehydrogenase (LDH) assay (Supplementary Fig. 2). The percentage of 1,4-NADH formed was then quantified by ^1^H NMR spectroscopy as a function of time (Fig. 3b, Supplementary Fig. 3). This experiment was performed for both a Pd black catalyst and a Pt-coated catalyst (Fig. 3a). The yield of 1,4-NADH after 3 h was ~26% with the Pd black catalyst, and ~67% with the Pt-coated catalyst (Fig. 3b). 1,6-NADH was the primary byproduct observed by ^1^H NMR (Supplementary Fig. 3b). 1,6-NADH is an inactive form of cofactor commonly observed as a byproduct within electrochemical and chemical regeneration. The faradaic efficiency for NADH formation was ~1%, indicating that the majority of hydrogen is lost through H_2_ generation at the surface of the membrane. This low faradaic efficiency is likely a result of the low quantity of NAD^+^ (0.015 mmol) relative to the high quantity of hydrogen permeating through the membrane (~4.7 mmol h^–1^). The faradaic efficiency could be increased to ~16% with a lower applied current density and increased NAD^+^ concentration (see Methods for experimental details). No NAD_2_ dimer was observed by ^1^H NMR (Supplementary Fig. 3) and ESI-MS (Supplementary Fig. 4)^32,33^. The higher rate and greater yield observed with the Pt-coated catalyst is consistent with previous studies identifying Pt as a highly active 1,4-NADH regeneration catalyst^34^. The higher selectivity of the Pt-coated catalyst to 1,4-NADH is likely due to the preferential binding of carbonyl to Pt^35^, which renders the C4’ position of NAD^+^ more electrophilic and accessible to surface-bound hydrogen. The mechanism of hydrogen permeation through Pd with subsequent migration onto Pt is discussed in depth in our previous work^35^. The turnover frequency of the Pt-coated catalyst was 1.5 µmol_1,4-NADH_ h^–1^ cm^–2^. Five cycles of NADH formation experiments showed high stability of the Pt-coated catalyst with <5% decrease in yield after 3 h (Supplementary Fig. 5).

We also examined 1,4-NADH formation with the Pt-coated catalyst as a function of pH (Fig. 3c). Basic conditions gave a greater yield of 1,4-NADH over the range of typical enzyme-relevant pH values (pH 6.5–9). This observation is consistent with previous studies that demonstrated that basic conditions mitigate acid-catalyzed decay pathways for 1,4-NADH^36,37^. We also observed that 20‒30% of 1,4-NADH decays in a buffer at both pH 6.5 and 7 at 37 ^°^C over 3 h in the absence of catalyst and applied current (Supplementary Fig. 6). This instability suggests the disparate yields of 1,4-NADH at different pH values is a result of 1,4-NADH degradation over the course of the 3 h experiment at lower pH values. Such degradation will not be a significant issue if the NADH generated is quickly consumed. It should also be noted that if a polar organic solvent such as dimethylsulfoxide (DMSO) was used in lieu of water, no NADH was formed (Supplementary Fig. 7). Our results demonstrate that the BioPMR can regenerate 1,4-NADH from aqueous NAD^+^ with high yield (67%) and minimal byproduct formation (Supplementary Fig. 3) using water electrolysis.

Scanning electron microscopy (SEM) images of the Pt-coated catalyst surface before (Supplementary Fig. 8a) and after five NADH generation experiments (Supplementary Fig. 8b) showed that the high-surface morphologies of the Pt-coated Pd catalyst were retained during five cycles of NADH regeneration. Top-view SEM images of the Pt-coated catalyst with an energy-dispersive X-ray spectroscopy (EDX) analysis showed a minimal change in atomic % Pt (Δ_Pt%_ ≈ 0.3%) and Pd (Δ_Pt%_ ≈ 1.5%), which suggested negligible degradation of the Pt-coated catalyst after five cycles of NADH regeneration (Supplementary Fig. 9).

**Fig. 4:**
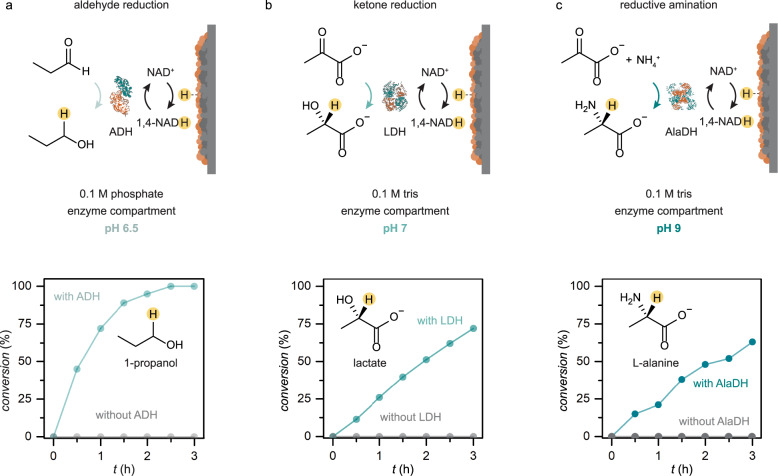
Performance of enzymatic reactions in the BioPMR at pH 6.5, 7, and 9. **a** Reduction of propionic aldehyde with alcohol dehydrogenase (ADH) in phosphate buffer at pH 6.5. **b** Reduction of pyruvic acid with lactate dehydrogenase (LDH) in tris buffer at pH 7. **c** Reductive amination of pyruvic acid with alanine dehydrogenase (AlaDH) in tris buffer at pH 9. Each enzymatic reaction mixture contains 1 mg/mL BSA.

We proceeded to study enzyme catalysis in the BioPMR with concurrent 1,4-NADH regeneration. Three different relevant enzymatic processes were examined: (i) aldehyde reduction (Fig. 4a); (ii) ketone reduction (Fig. 4b), and; (iii) reductive amination (Fig. 4c). These enzymatic reactions were chosen because each has a different optimum operating pH^38–40^. Each reaction mixture consisted of 5 units of enzyme and 1 mg/mL bovine serum albumin (BSA) with 0.1 M of the appropriate buffer in the enzyme compartment. The electrochemical compartment was filled with 1 M H_2_SO_4_. All enzymatic studies were performed at 37  ^°^C for 3 h with an applied current density of ‒50 mA cm^–2^ (*V*_avg_ = ‒0.3 ± 0.05 V vs. SHE, Supplementary Fig. 10a–c). Product conversion was tracked as a function of time by ^1^H NMR (Fig. 4, Supplementary Figs. 11–13). All enzymes show high yields (65–100%) and conversion rates for their respective substrates. Control experiments with no added NAD^+^ and no enzyme confirmed that the NADH-dependent enzymes were responsible for product formation (Fig. 4). We also performed the enzymatic aldehyde reduction with 1:5 and 1:25 equivalents of substrate relative to NAD^+^, and observed ~100% conversion in 4 h and >95% conversion 24 h, demonstrating a turnover number of ~2.2 h^–1^ (Supplementary Fig. 14).

**Fig. 5:**
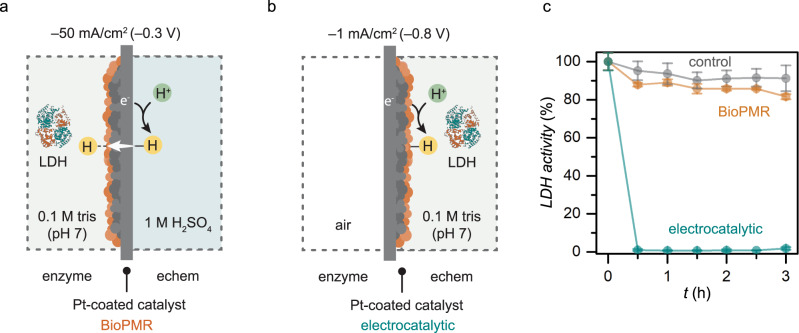
Enzyme stability for BioPMR and electrocatalytic regeneration conditions. Cell setup for **a** BioPMR regeneration conditions and **b** conventional electrocatalytic regeneration conditions. **c** LDH activity as a function of time for both setups. Each enzyme solution contains 1 mg/mL BSA. Error bars represent the standard deviation of triplicate measurements. The center point of each error bar represents the mean of the triplicate measurements.

We hypothesized that the isolation of the enzyme from the electrochemical process in the BioPMR would increase the enzyme stability. We tested this hypothesis by performing two experiments with LDH under similar conditions, one in the BioPMR (Fig. 5a) and one under conventional electrocatalytic regeneration conditions (Fig. 5b). Both experiments were performed under electrolysis with LDH in 0.1 M tris (pH 7). The appropriate potential (–0.8 V vs. SHE) for NAD^+^ reduction in the electrocatalytic experiment was identified by cyclic voltammetry (CV, Supplementary Fig. 15). The activity of LDH was lost after 30 min for electrocatalytic regeneration conditions while BioPMR conditions retained activity comparable to the control in which no electrolysis was performed (Fig. 5c).

**Fig. 6:**
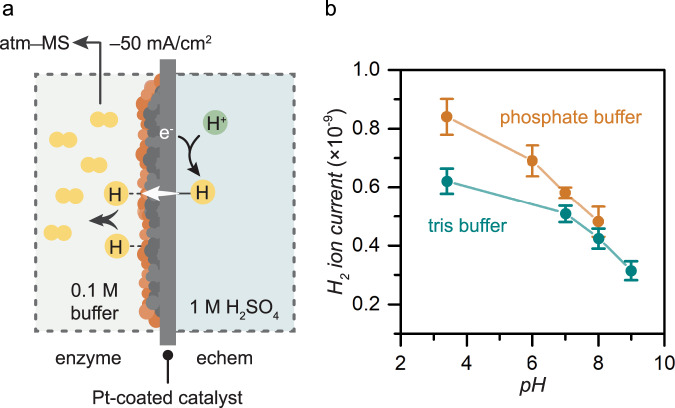
pH-dependent hydrogen permeation in the BioPMR. **a** Cell setup. **b** H_2_ gas evolution at steady-state with a Pt-coated catalyst as a function of pH for tris (teal) and phosphate (orange) buffer. Error bars represent the standard deviation of triplicate measurements. The center point of each error bar represents the mean of the triplicate measurements.

We conducted pH-dependent hydrogen permeation studies to probe the mechanism of NADH regeneration in the BioPMR (Fig. 6). We hypothesized that the mechanism of formation of the reduced product (NADH) involved disproportionation of H atoms (i.e., H· + H· → H^+^ + H^−^) on the catalyst surface. These charged H species (i.e., H^+^ + H^-^) will desorb as H_2_ (i.e., H^+^ + H^–^ → H_2(g)_) if H^−^ does not react with NAD^+^. Our previous study demonstrated that the amount of H_2(g)_ formed on the Pt-coated Pd catalyst in the enzyme compartment is governed by the desorption process^34^. This desorption process would result in a pH-dependence of the hydrogen generation in the enzyme compartment while H_2_ desorption that does not involve disproportionation of H atoms (i.e., H· + H· → H_2(g)_) would not be pH-dependent.

A BioPMR cell was connected to an atmospheric mass spectrometer (atm–MS), with gas sampling of the enzyme compartment (Fig. 6a). The cell was filled with electrolyte (1 M H_2_SO_4_) in the electrochemical compartment and buffer only (1 M tris or phosphate) in the enzyme compartment. Electrolysis was performed (*I*_applied_ = –50 mA cm^–2^) and the amount of H_2_ gas generated at the catalyst surface was allowed to reach steady state (~1 h). We confirmed that the H_2_ generated in the chemical compartment was stable (0.76 mmol cm^–2^ h^–1^) after reaching steady-state (Supplementary Fig. 16c). This stability was retained even if the foil was repeatedly used to regenerate NAD^+^ (Supplementary Fig. 16b). A series of these measurements were performed at sequential buffer pH values (pH_tris_ = 3.4, 7, 8, 9; pH_phosphate_ = 3.4, 6, 7, 8). We observed that lower pH values resulted in higher H_2_ gas generation for both tris and phosphate buffer (Fig. 6b). A detailed discussion of the results is provided below. It should be noted that the pH 3.4 data points extend outside of the buffering pH range.

## Discussion

This work demonstrates that the BioPMR can be used to: (i) electrochemically regenerate NADH from NAD^+^, and; (ii) perform NADH regeneration in parallel with enzyme catalysis. The BioPMR has several advantages over conventional enzymatic and electrochemical cofactor regeneration methods. First, the BioPMR does not require any secondary enzymes, co-substrates, or immobilized mediators. Second, the only major waste product in the BioPMR is oxygen gas generated at the anode. Both advantages are in contrast to formate and glucose dehydrogenase systems that require separation of water-soluble co-substrates and byproducts^13,14^, or electrochemical cofactor regeneration systems that often require immobilized mediators to suppress inactive NAD_2_ formation^22^. Both advantages minimize complexity and the purification steps required for isolation of the desired product.

There are additional advantages of the BioPMR that stem from the separation of the electrochemical and enzymatic processes. Typical electrochemical cofactor regeneration systems with both processes occurring in the same reaction mixture necessitate compromise with regard to the ideal operating conditions (e.g., pH, temperature, ionic strength, and catalyst choice) for each process. Enzyme inactivation or denaturation can also occur from the electric field or resistive heating at the electrodes in these systems^22^. In the BioPMR, the Pd foil separates the two compartments and enables the optimal conditions for each process to be used while also isolating the enzyme from the electric field of the cell. Moreover, the separation of the BioPMR enables a lower overpotential for NADH formation at biologically relevant pH values (pH 6.5–9.0) relative to a typical electrochemical cofactor regeneration system (–0.3 V vs SHE (Supplementary Fig. 10) and –0.6 to –1.7 V vs. SHE^41,42^, respectively). The operating voltage also showed no change for >10 h of electrolysis (Supplementary Fig. 17). In summary, the BioPMR can perform a wide range of NADH-dependent enzymatic transformations on different reagents under ideal operating conditions with water and electricity as the primary inputs and benign gasses as the primary outputs.

NADH formation from NAD^+^ proceeds through one of the following mechanisms: (a) electron transfer and proton transfer (2e^–^ + H^+^); (b) electron transfer and hydrogen transfer (e^–^ and H), or; (c) hydride transfer (H^–^). Electrocatalytic NADH generation follows (a) or (b)^22^. NADH formation in the BioPMR is unlikely to undergo mechanisms (a) and (b) because the surface of the catalyst is isolated from the electrochemical potential of the cell (Fig. 1c), thus any pathway involving direct e^–^ transfer is unlikely. This assertion is also supported by the absence of NAD_2_ formation in the BioPMR, which occurs following direct electron transfer.

**Fig. 7:**
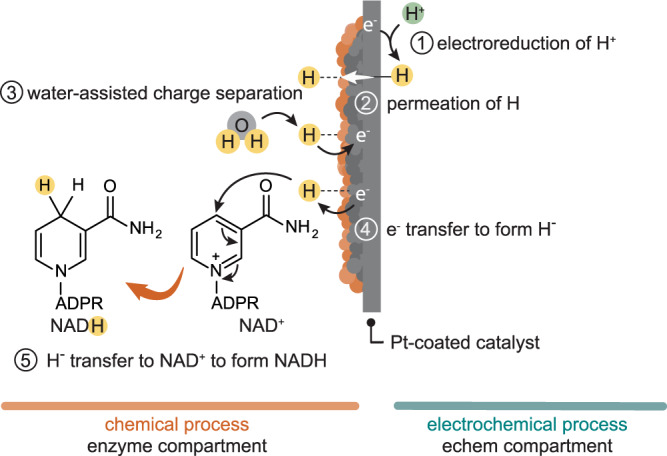
Proposed water-assisted NADH formation mechanism. (1) Protons are reduced at the Pd foil to form atomic hydrogen; (2) atomic hydrogen permeates through the Pd foil to form surface-boundhydrogen; (3) surface-bound hydrogen undergoes water-assisted charge separation, producing hydronium and a reduced metal surface; (4) electron transfer from the reduced metal to surface-bound hydrogen forms hydride; (5) hydride transfers to NAD^+^, forming 1,4-NADH.

The presence of surface-bound hydride is supported by the pH-dependence of H_2_ gas generation experiments. These experiments demonstrate that higher H^+^ concentrations result in faster H_2_ gas generation. We conjecture that the presence of charged hydrogen species (i.e., H^+^ and H^−^) on the catalyst surface is responsible for the accelerated H_2_ gas generation at low pH. This acceleration would not be expected if the desorption-limited hydrogen evolution^35^ was solely occurring through charge neutral processes (e.g., H· + H· → H_2_). The formation of charged hydrogen species on precious metal surfaces has been previously demonstrated^43^. This process occurs through water binding with surface hydrogen followed by heterolytic cleavage of the Pt–H bond, which ultimately forms H_3_O^+^ and transfers an e^-^ to the Pt bulk. Such a mechanism can be conceptualized as a reverse Volmer process (H^+^ + e^–^ → H). We confirmed that water is necessary for NADH regeneration to occur by performing comparable experiments in DMSO (Supplementary Fig. 7). We propose a mechanism here (Fig. 7) where the formation of hydride occurs through water-assisted disproportionation of H atoms (e.g., H· + H· → H^+^ + H^–^) on the Pt-coated catalyst.

In conclusion, we demonstrate a strategy for performing NADH-driven enzymatic reactions. This platform enables 1,4-NADH to be electrochemically regenerated without the formation of inactive NAD_2_. The system uses a Pd foil as both a cathode and selective membrane that enables both enzymatic and electrolytic processes to be physically separated by the membrane. This architecture increases the stability of the enzyme relative to conventional electrochemical NADH regeneration methods and requires a lower overpotential NAD^+^/NADH conversion at physiological pH (6.5–9.0). A variety of enzymatic reactions are performed using the BioPMR at high yield under their optimum enzyme pH (Fig. 4) conditions using in situ regenerated 1,4-NADH. We also show that this system regenerates 1,4-NADH through direct hydride transfer to NAD^+^. This hydride transfer likely involves water-assisted charge separation of H atoms at the catalyst surface. The BioPMR is a method for 1,4-NADH regeneration that with further study (e.g., Pd membranes with immobilized enzyme and cofactor for faster reaction rates^44^) could result in an expansion of the use of NADH-dependent enzymes for industrial chemical production.

## Methods

### Materials

Pd (99.95%) was obtained from Silver Gold Bull. β-Nicotinamide adenine dinucleotide hydrate (≥99%), β-Nicotinamide adenine dinucleotide, reduced disodium salt hydrate (≥97%, HPLC), propionic aldehyde (reagent grade, 97%), ammonium chloride, bovine serum albumin, *L*-lactic dehydrogenase from rabbit muscle, alcohol dehydrogenase from Saccharomyces cerevisiae, *L*-alanine dehydrogenase from Bacillus subtilis, sulfuric acid (95.0–98.0%), H_2_O_2_ solution (30 wt.% in H_2_O) and dimethylsulfone (quantitative NMR standard, TraceCERT) were purchased from Sigma Aldrich and used as received. Sodium pyruvate (≥99%) was purchased from Fisher Scientific. 68–70% Nitric acid was obtained from VWR. Pt gauze (52 mesh, 99.9%) and Pt wire (0.5 mm, 99.95%) were obtained from Alfa Aesar. Ag/AgCl reference electrodes (RE5B) were purchased from BASi.

### Cell design

The three cell cores (Fig. 2) are printed by a stereolithography 3D printer. The cores are composed of high-temperature resistant resin (Formlabs proprietary resin). The membrane is sealed between the cores by Viton ring gaskets, and the assembly is clamped together by two waterjet cut ¼ in thick aluminum end plates connected by four M4 stainless steel bolts. Quick-turn polycarbonate couplings (¼−28 in) were used to feed gas inlet/outlet ports on the enzyme cell (threads were printed in the core).

### Electrochemistry

A Metrohm Autolab PGSTAT302N/PGSTAT204M potentiostat was used for electrochemical experiments. Electrochemical data was analyzed both with Metrohm Autolab NOVA (Version 2.1) and Origin (Version 9.3) palladium foil was fitted between the enzyme and cathode electrochemical compartment to assemble the electrocatalytic palladium membrane reactor. Viton ring gaskets were used to seal the palladium foil in place and prevent leaking. The enzyme compartment was filled with 10 mL of tris or phosphate buffer. The electrochemical compartment was filled with 8 mL of 1 M H_2_SO_4_. A Ag/AgCl reference electrode (3.0 M NaCl) and a Pt mesh counter electrode were housed in the electrochemical compartment. All potentials are reported versus SCE unless stated otherwise. Electrolysis was conducted galvanostatically at –50 mA cm^–2^ (*V*_avg_ = –0.3 V vs. SCE) for the BioPMR experiments. The uncompensated resistance was 1–2 Ω for all experiments, and no IR correction was used. The thickness of the foil was 25 μm and the geometric surface area of the foil was 2.5 cm^2^. Cyclic voltammetry (CV) measurements were performed under an Ar atmosphere with a Pt-coated Pd working electrode, a Ag/AgCl reference electrode, a Pt mesh counter electrode, and a scan rate of 250 mV/s. The faradaic efficiency (FE) was calculated assuming a reaction stoichiometry of 2 H· per H^−^ equivalent:

1 $${{{{{\rm{FE}}}}}}={({mols}{NADH})}/{(0.5\cdot {{{{{{\rm{I}}}}}}}_{{applied}}\cdot t \cdot {{{{{{\rm{F}}}}}}}^{-1})}.$$

### Pd foil and Pd black catalyst preparation

Pd foils were rolled from a palladium wafer bar to a thickness of 25 μm. The thickness of the foil was measured by a Mitutoyo digital micrometer to an accuracy of ±1 μm. The freshly rolled foil was annealed in Ar at 850 °C for 1.5 h and stored in air. Prior to use, the annealed foils were cleaned by soaking the foil in a 0.5:0.5:1 (by volume) conc. HNO_3_:H_2_O:30% H_2_O_2_ for ~45 min, and subsequently cleaned with Milli-Q ultrapure water. The Pd black catalyst was then electroplated onto the surface. The cell was assembled as described above, except the cathode compartment was filled with an electrodeposition solution (15.9 mM PdCl_2_ in 1 M HCl) in lieu of the 1 M H_2_SO_4_ electrolyte. The solution was stirred at a constant rate (400 rpm) and a voltage of –0.1 V vs. SCE was applied to the Pd foil working electrode to reduce the Pd ions in solution. The electrodeposition was stopped when 18.5 C of charge (7.38 C/cm^2^) had been passed. The mass loading for all electrodepositions was ~10 mg. The sputter-deposited Pt-coated samples were within error of this mass loading. The foils were then thoroughly rinsed with Milli-Q ultrapure water, covered in a 4” diameter petri dish to maintain cleanliness, and stored in ambient conditions.

### Pt-coated membrane preparation

The catalyst-coated membranes were prepared by sputter deposition. The electrodeposited palladium foils were placed in a Leica EM MED020 coating system at a working distance of 6 cm, the chamber was sealed, and a vacuum applied to achieve a base pressure of 1 × 10^–4^ mbar. After the base pressure was reached, argon was continuously flowed into the chamber to maintain a pressure of 1 $\times$10^–2^ mbar, the plasma was ignited, and voltage was adjusted to maintain a constant sputter current of 30 mA. Following a 30 s presputter, the target shutter was opened and 10-nm platinum was sputtered onto the electrodeposited palladium catalyst. The sputtering rate was 0.2 nm/s, as determined by in situ quartz crystal microbalance. The foils were used for NADH regeneration and enzymatic reduction/reductive amination experiments without any further processing. The catalyst-coated membranes were used for up to 20 reactions. The entire catalyst layer of Pt-coated Pd black was removed with concentrated nitric acid, then the foil was cleaned (described above), replated, and reused. Each palladium foil was used for ~5 cleaning cycles before being discarded.

### Buffer preparation

Phosphate buffers were prepared by dissolving the appropriate amounts of disodium and monosodium phosphate salts in deionized water. Tris (tris(hydroxymethyl)aminomethane) buffer was prepared by dissolving tris salts in deionized water. The pH for these buffers was then adjusted with the addition of the appropriate amounts of 1 M aqueous solutions of NaOH and/or HCl.

### NADH generation

NADH regeneration was performed in a 0.1 M phosphate or tris buffer at pH 6.5, 7, and 9. All reactions were carried out in an oven at 37  ^°^C under Ar. Enzyme compartment of the 3D-printed cell with a magnetic stir bar was filled with 1.5 mM NAD^+^ and 10 mg bovine serum albumin (BSA) in buffer (10 mL) or dry DMSO. 1 M H_2_SO_4_ electrolyte solution (8 mL) was added to an electrochemical compartment and a constant reductive current of –50 mA was applied for 3 h. NADH reaction mixture was stirred at a constant rate (400 rpm) throughout the experiment. Reaction aliquots were sampled every 0.5 h to monitor the reaction progression of NADH generation by UV/Vis and ^1^H NMR spectroscopy. UV/Vis absorption spectra of reaction mixture were collected by using a Jasco J-815 spectrometer without dilution. All measurements were performed at room temperature with the sample perpendicular to the light path. A tris buffer blank was used as the baseline measurement. Solution temperature and pH were checked before and after the reaction. Electrospray mass spectrometry was recorded with a Waters ZQ mass spectrometer equipped with ESI ion source without sample dilution. The faradaic efficiency could be increased from ~1% to ~16% by decreasing the current density (–50 mA/cm^2^ to –5 mA/cm^2^) and increasing the NAD^+^ concentration (1.5 mM to 15 mM).

### Scanning electron microscopy (SEM) and X-ray energy-dispersive spectroscopy (EDX)

Scanning Electron Microscope (SEM) and energy-dispersive X-ray spectroscopy (EDX) experiments were performed using a Helios NanoLab 650 Focused Ion Beam SEM. EDX data was acquired from 600 μm^2^ regions over 50 s at 10 kV accelerating voltage and 120 nA.

### Enzyme catalysis

All enzyme-catalyzed reactions were carried out in the oven at 37 ^°^C under Ar. The enzyme compartment contained a magnetic stir bar and was filled with 1.5 mM of NAD^+^, 10 mg BSA, 1.5 mM of substrates (propionic aldehyde for aldehyde reduction, sodium pyruvate for ketone reduction, pyruvate and 25 mM NH_4_Cl for reductive amination), and 5 units of the corresponding enzyme (alcohol dehydrogenase for aldehyde reduction, lactate dehydrogenase for ketone reduction, and *L*-alanine dehydrogenase for reductive amination) in 10 mL of the buffer. Alcohol reduction was performed in 0.1 M phosphate buffer at pH 6.5; ketone reduction was performed in 0.1 M tris buffer at pH 7; reductive amination was performed in 0.1 M tris buffer at pH 9. 1 M H_2_SO_4_ electrolyte solution (8 mL) was added to the electrochemical compartment and a constant reductive current of –50 mA was applied for 3 h. The reaction mixture was stirred at a constant rate (400 rpm) throughout the experiment. Reaction aliquots were sampled every 0.5 h to monitor the reaction progression of product formation by ^1^H NMR spectroscopy. Aldehyde reduction was also performed with 1:5 and 1:25 mol equivalent of NAD^+^ (1.5 mM) and propionic aldehyde (7.5 mM or 37.5 mM) with 10 or 30 units of ADH in 0.1 mM phosphate buffer at pH 6.5, respectively.

### Product quantification

Proton nuclear magnetic resonance (^1^H NMR) was used for product quantification. 460 μL of the reaction mixture (1.5 mM) and 40 μL 0.2 M dimethylsulfone internal standard in D_2_O were added to an NMR tube. ^1^H NMR spectra were acquired on a Bruker Avance 400dir, or 400inv spectrometer at 298 K. Relative concentrations were determined by comparing the integrated signal of the methyl group of dimethylsulfone.

### Enzyme activity assay

All enzyme stability experiments were carried out in an oven at 37  ^°^C under Ar. 10 uL (~245 units) LDH and 30 mg BSA in 30 mL Tris buffer at pH 7 were prepared and divided into 3×10 mL solutions. For the BioPMR platform, the enzyme compartment was filled with an LDH solution and a stir bar. 1 M H_2_SO_4_ electrolyte solution (8 mL) was added to the electrochemical compartment and a constant reductive current of –50 mA (*V*_ave_ = –0.3 V vs. SCE) was applied for 3 h. For the electrocatalytic platform, the enzyme compartment was empty, and the Pt-coated side of the membrane was facing the electrochemical compartment. LDH solution was added to the electrochemical compartment and a constant potential of –0.8 V vs. SCE was applied for 3 h. The LDH solution was stirred at a constant rate (400 rpm) throughout the experiment. Reaction aliquots were sampled every 0.5 h to monitor the LDH stability in each platform. These samples were then diluted 100 times in the assay buffer (0.1 M Tris buffer pH 7, 2 mM NADH) and incubated for 2 min. The reactions were then initiated by the addition of sodium pyruvate (final concentration of 3.5 mM) and the rate of each reaction was determined based on the rate of the consumption of NADH, by monitoring the absorbance at 340 nm. The concentration of the remaining active enzyme after each time point was thus quantified, since the rate of these reactions are directly proportional to the enzyme concentration under these conditions. All the assays were performed in triplicate and in 96-well half-area plate [Corning] and monitored with a Synergy H1 plate reader [BioTek].

### H_2_ gas permeation experiment

An ESS CatalySys atmospheric mass spectrometer was used for pH-dependent hydrogen permeation experiments. Hydrogen permeation experiments were conducted with 1 M H_2_SO_4_ in the electrochemical compartment and phosphate or tris buffer at their buffering pH in the enzyme compartment. H_2_ generation outside of their buffering pH (pH 3.4) was also measured to examine if the pH-dependency extends outside of their buffering pH. The palladium foil was placed between the electrochemical and enzyme compartments with the Pt-coated surface facing into the enzyme compartment and a constant reductive current of –50 mA was applied. The production of gaseous H_2_ (2 m/z) in the enzyme compartment with constant stirring was monitored by atmospheric mass spectrometry (atm–MS) with a flow rate of 10 mL/min into the instrument. The ion current value was taken once the signal had equilibrated (~1 h). The average value for at least three experiments is reported, with error bars representing one standard deviation of the mean. The hydrogen diffusion at steady-state was calculated as follows:

2 $$\left[{{{Hydrogen}}_{c{hem}}}/{\left({{Hydrogen}}_{c{hem}}+{{Hydrogen}}_{ec{hem}}\right)}\right]\left[{I}_{a{pplied}}\right.\\ \left.\times \left(3600{{{{{\rm{s}}}}}}/1{{{{{\rm{h}}}}}}\right)\times \left({1}/{F}\right)\times \left({{mol}}_{H2}/2{{mol}}_{{e}^{-}}\right)\times \left({1}/{{{SA}}_{g{eometric}}}\right)\right].$$

Where *Hydrogen*_*chem*_ is the mols of hydrogen evolved in the chem compartment, *Hydrogen*_*echem*_ is the mols of hydrogen evolved in the echem compartment, *I*_*applied*_ is the total current in A, F is Faraday’s constant, and *SA*_*geometric*_ is the geometric surface area of the membrane in cm^–2^. For steady-state H_2_ flux measurements, electrolysis was performed at –50 mA/cm^2^ for 1 h to reach steady-state conditions. At 1 h, atm–MS measurements were performed and the H_2_ ion current was recorded for 10 h.

### Reporting summary

Further information on research design is available in the Nature Portfolio Reporting Summary linked to this article.

## Supplementary information

Supplementary Information

Reporting Summary

## Data Availability

The data supporting the findings in this study are available either within the paper or its Supplementary Information, or from the corresponding author on request.
