# Supplementary material for: Bioelectrocatalysis with a palladium membrane reactor
Source: Nat Commun. 2023 Mar 31;14:1814. doi: 10.1038/s41467-023-37257-7 (PMC10066381; doi:10.1038/s41467-023-37257-7)
Supplement: Supplementary file 1 — Supplementary Information [file 41467_2023_37257_MOESM1_ESM.pdf]

Supplementary Information

Bioelectrocatalysis with a palladium membrane reactor

Aiko Kurimoto,[1] Seyed A. Nasseri,[1] Camden Hunt,[1,2] Mike Rooney,[1] David J. Dvorak,[2] Natalie E. LeSage,[1] Ryan P. Jansonius,[1] Stephen G. Withers,[1] Curtis P. Berlinguette[1,2,3,4*]

[1]Department of Chemistry, The University of British Columbia, 2036 Main Mall, Vancouver, British Columbia, V6T 1Z1, Canada.

[2]Stewart Blusson Quantum Matter Institute, The University of British Columbia, 2355 East Mall, Vancouver, British Columbia, V6T 1Z4, Canada.

[3]Department of Chemical and Biological Engineering, The University of British Columbia, 2360 East Mall, Vancouver, British Columbia, V6Y 1Z3, Canada.

[4]Canadian Institute for Advanced Research (CIFAR), 661 University Avenue, Toronto, M5G 1M1, Ontario, Canada.

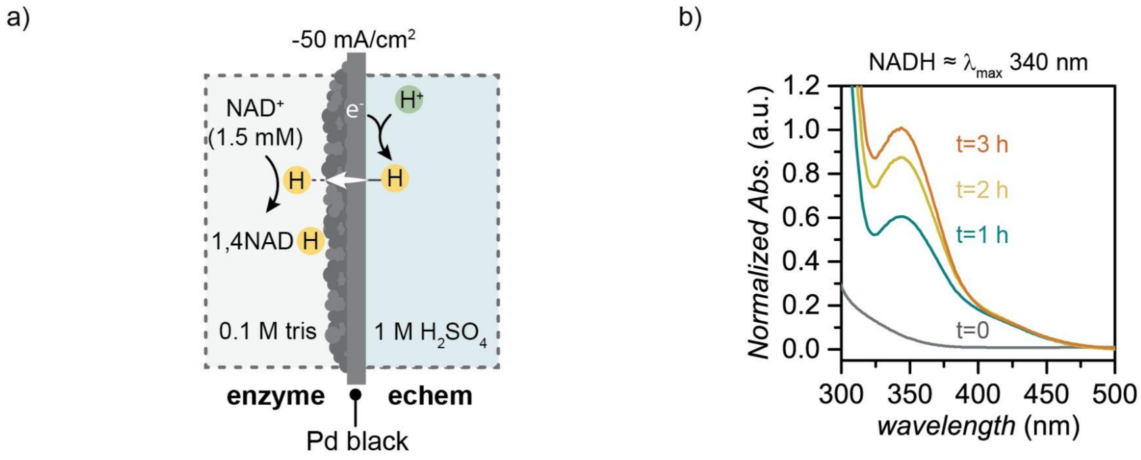

Supplementary Figure 1. NADH generation from NAD^+ in tris at pH 9 with Pd black catalyst. a) Cell setup and idealized process. b) UV/Vis absorption spectra of NADH generation as a function of time.

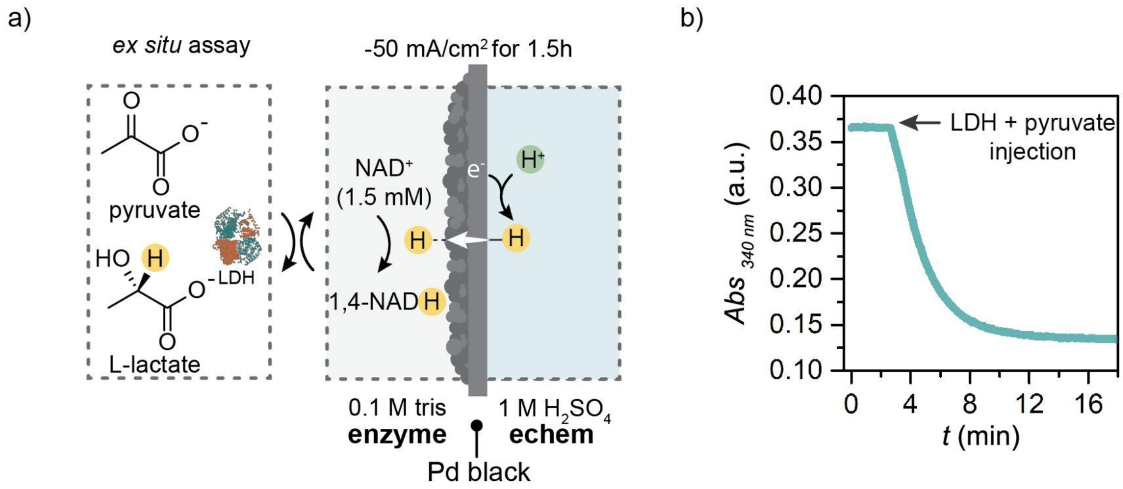

Supplementary Figure 2. NADH generation from NAD^+ in tris buffer at pH 9 with Pd black catalyst. a) Cell setup and idealized process. b) *Ex-situ* LDH assay study.

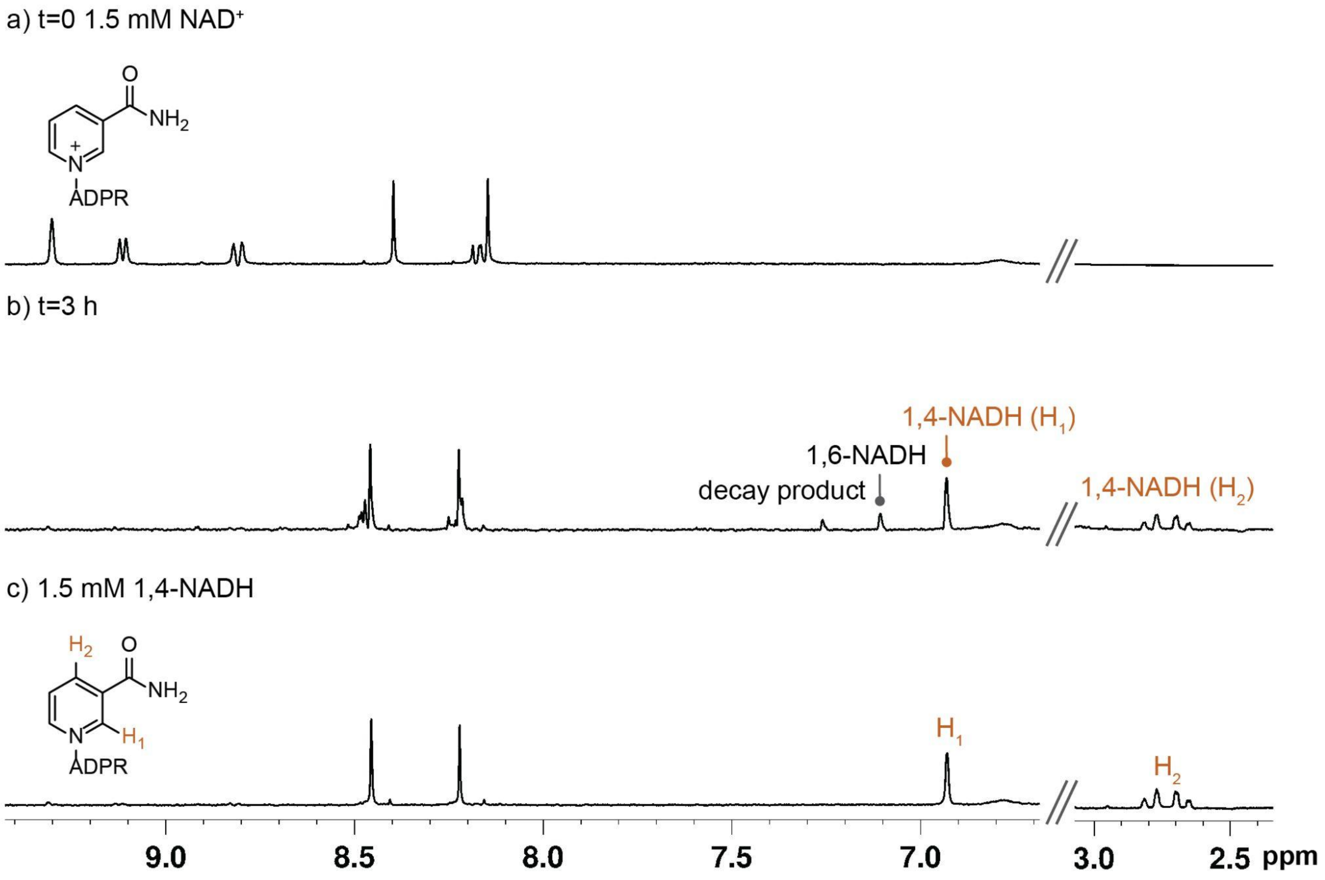

Supplementary Figure 3. ^{1}H NMR spectra of NADH generation of 1.5 mM NAD^+ in tris buffer at pH 9 with Pt-coated catalyst at 50 mA·cm^{-2}. a) The initial spectrum. b) The spectrum after 3 h. c) The control 1,4-NADH spectrum.

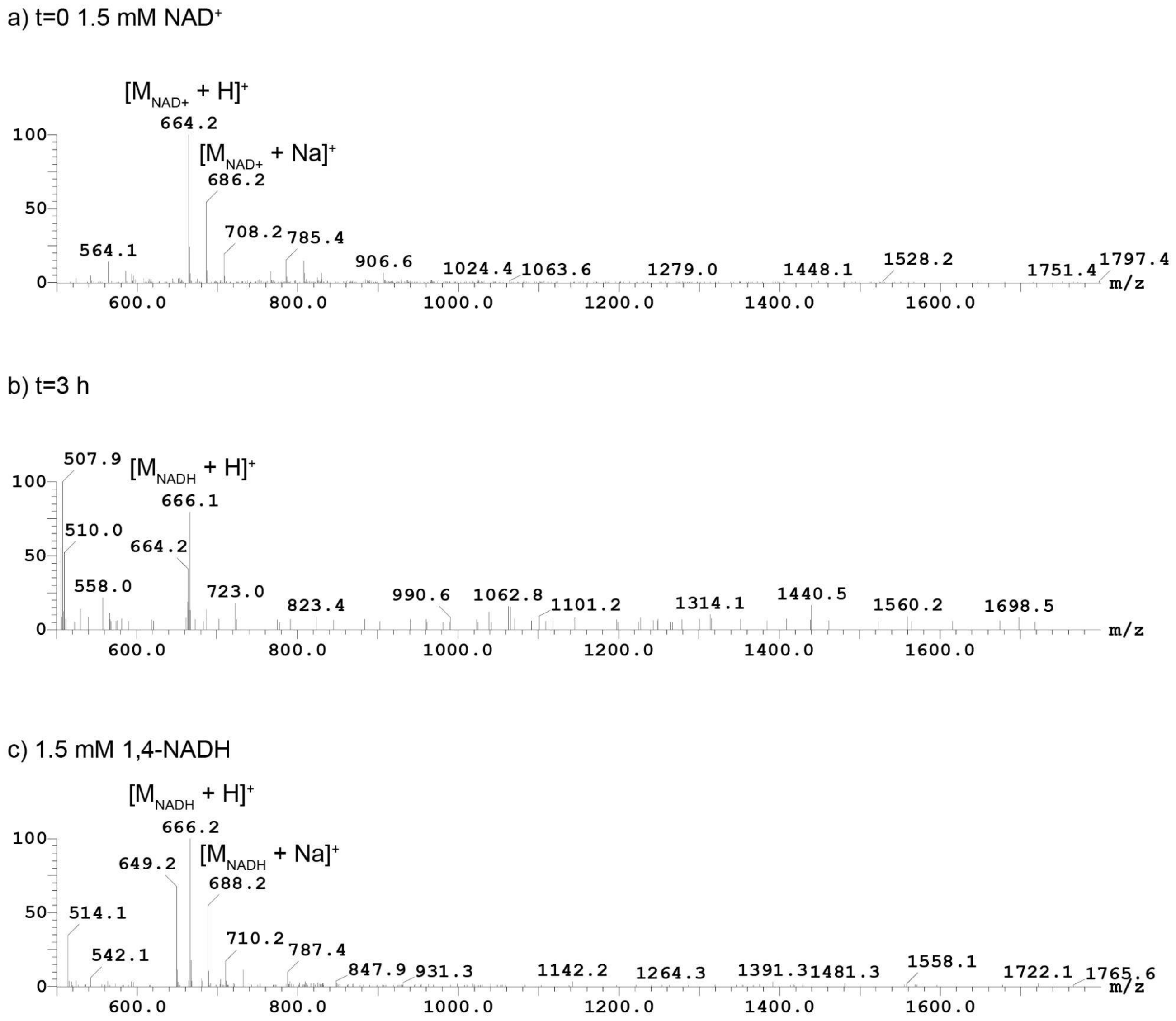

Supplementary Figure 4. ESI-MS of NADH generation of 1.5 mM NAD^+ in tris buffer at pH 9 with Pt-coated catalyst at 50 mA·cm^{-2}. a) The initial spectrum. b) The spectrum after 3 h. c) The control 1,4-NADH spectrum.

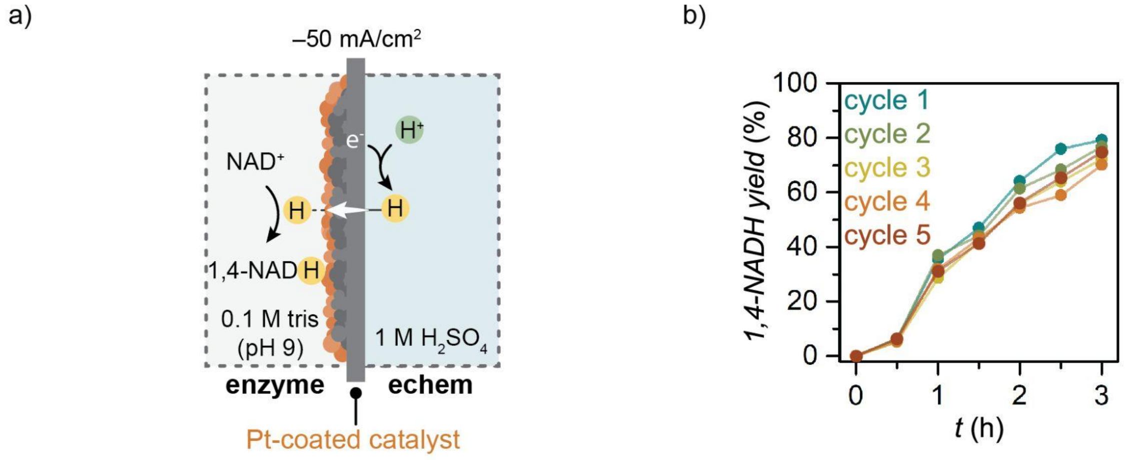

Supplementary Figure 5. Reusability of Pt-coated catalyst. a) Cell setup and idealized process. b) 1,4-NADH formation as a function of time during 5 individual cycles using the same foil.

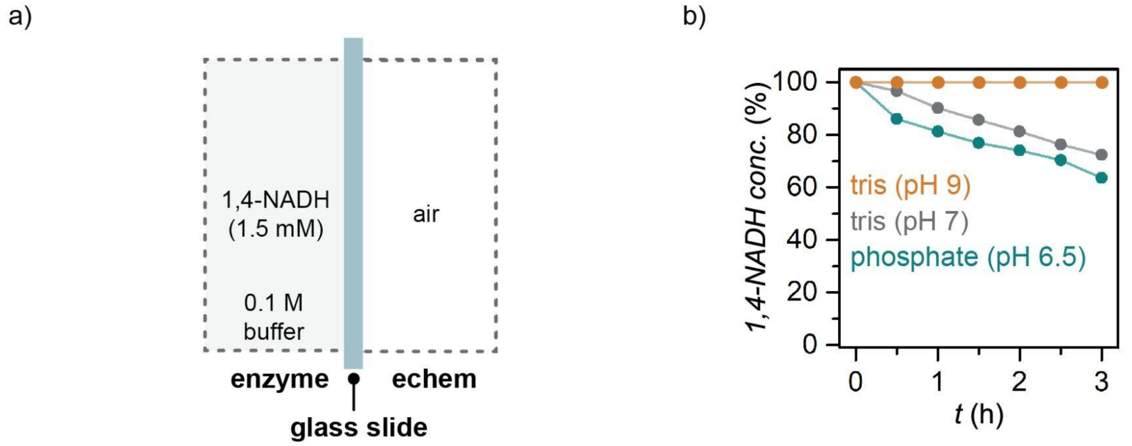

Supplementary Figure 6. NADH stability as a function of pH. a) Cell setup. b) Residual 1,4-NADH concentration at pH 6.5 (phosphate buffer), pH 7 (tris buffer), and pH 9 (tris buffer) as a function of time.

a)

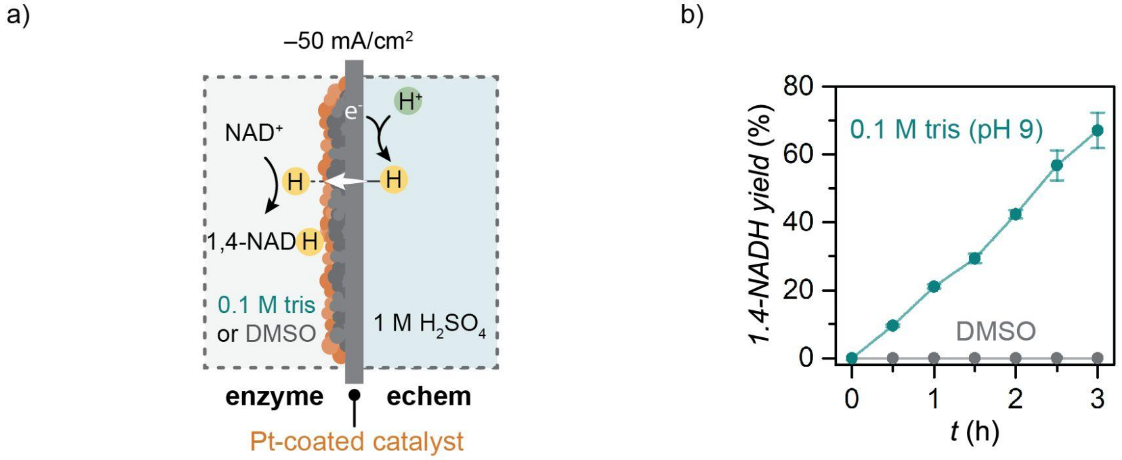

Supplementary Figure 7. NADH generation from NAD^+ in both tris buffer (pH 9) and DMSO. a) Cell setup and idealized process. b) 1,4-NADH formation as a function of time. Error bars represent the standard deviation of triplicate measurements on different foils. The center point of each error bar represents the mean of the triplicate measurements.

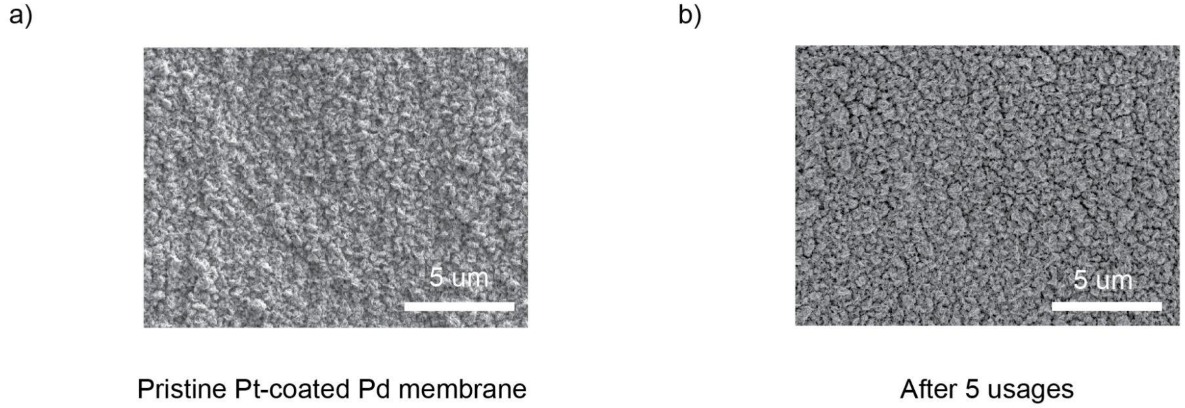

Supplementary Figure 8. Top-view SEM images of Pt-coated Pd membrane. a) before and b) after 5 times used for NADH regeneration experiments. SEM images were obtained at 6500x magnification. Micrographs were obtained in duplicate on two different foils with no discernible difference.

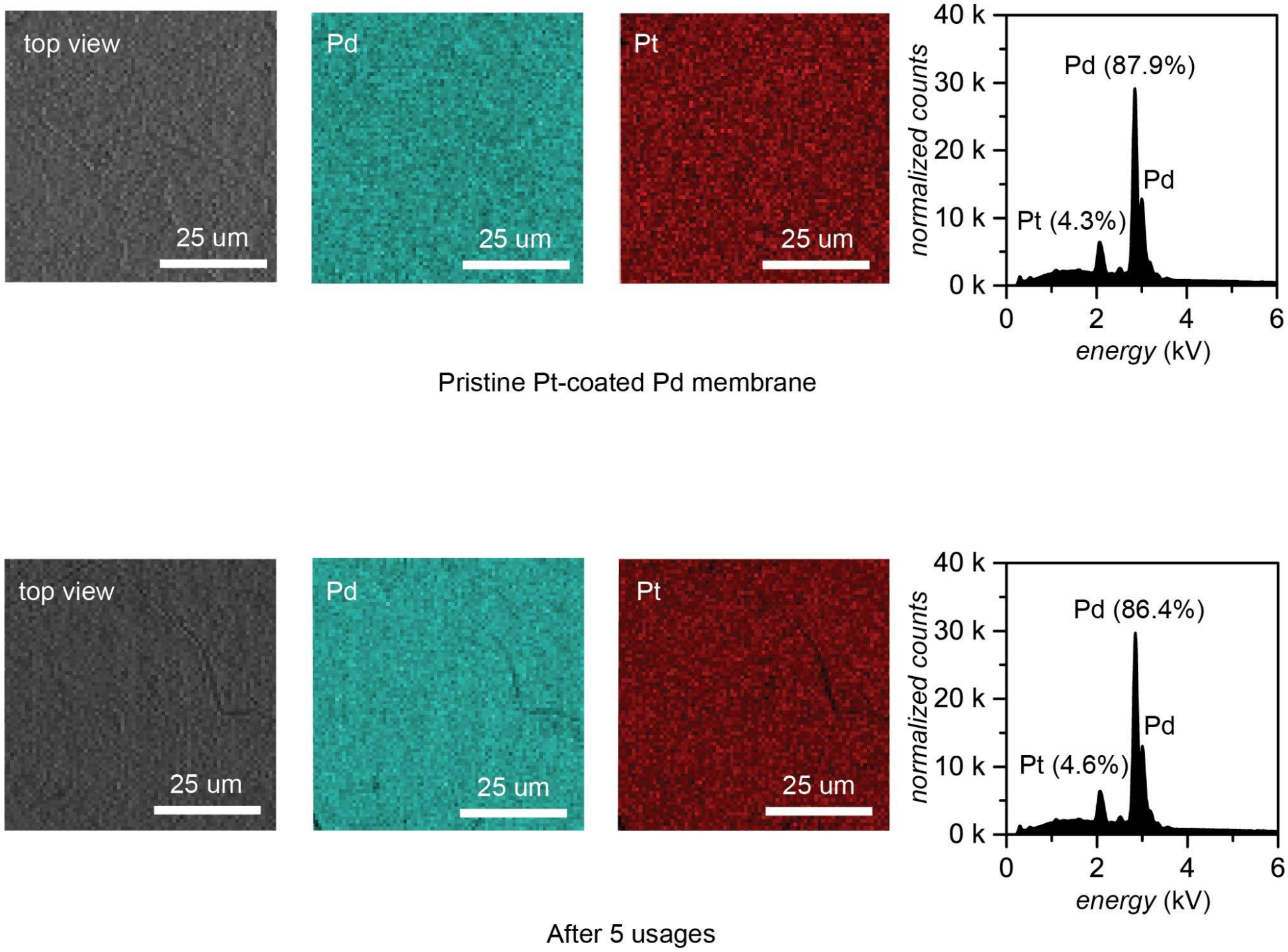

Supplementary Figure 9. SEM and EDX analyses of Pt-coated Pd membrane. Top-view before (top) and after 5 times used (bottom) for NADH regeneration experiments. Elemental analyses (measured using EDX) corresponding to the SEM micrograph are shown for palladium and platinum. Micrographs were obtained in duplicate on two different foils with no discernible difference.

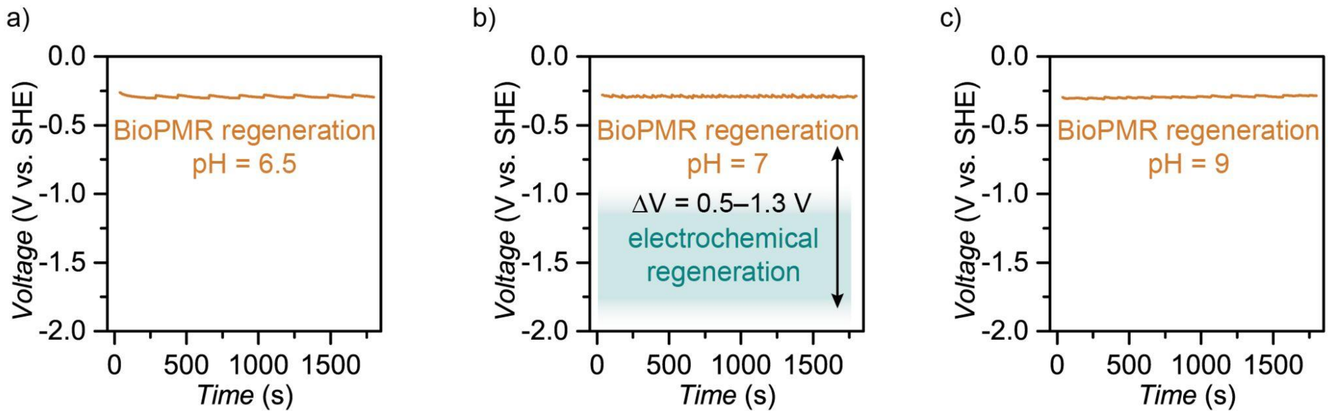

Supplementary Figure 10. The voltage range for NADH regeneration in the BioPMR. a) pH 6.5, b) pH 7, and c) pH 9. The voltage range for typical electrochemical systems are also shown (teal).

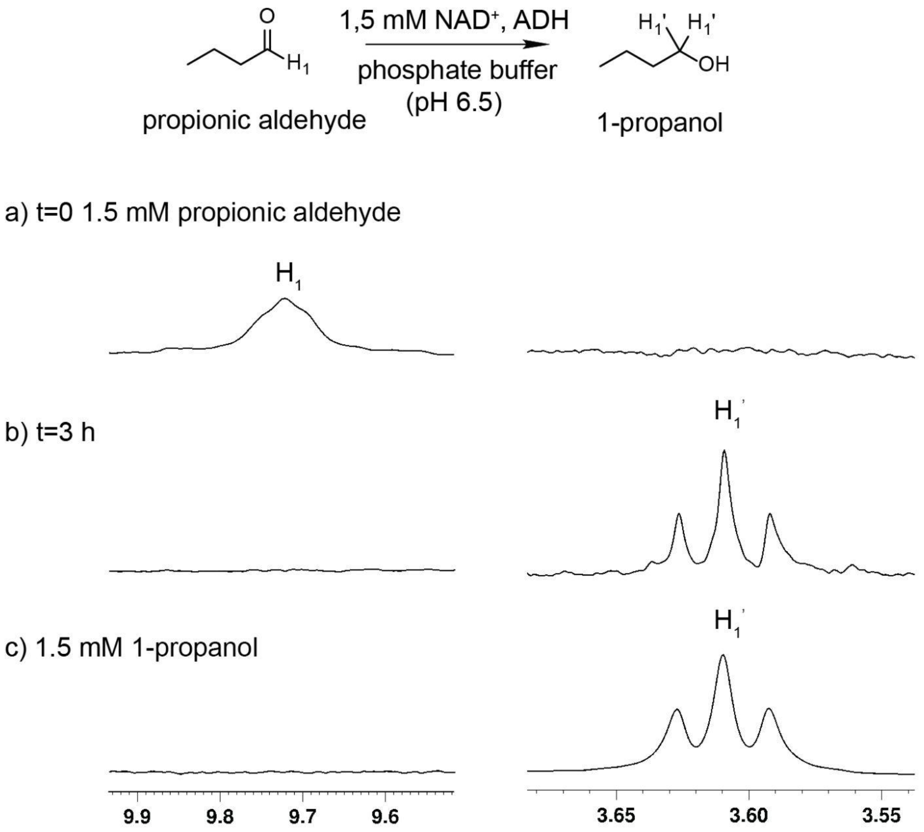

Supplementary Figure 11. ^{1}H NMR spectra of 1-propanol formation from propionic aldehyde. a) The initial spectrum. b) The spectrum after 3 h. c) The control 1-propanol spectrum.

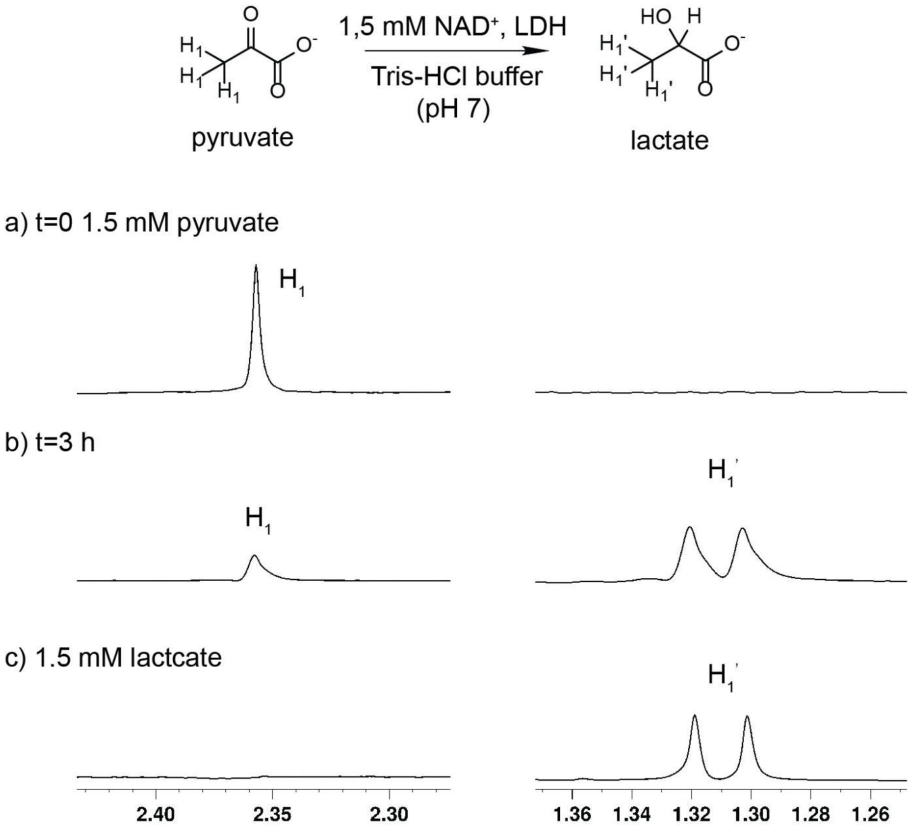

Supplementary Figure 12. ^{1}H NMR spectra of lactate formation from pyruvate. a) The initial spectrum. b) The spectrum after 3 h. c) The control lactate spectrum.

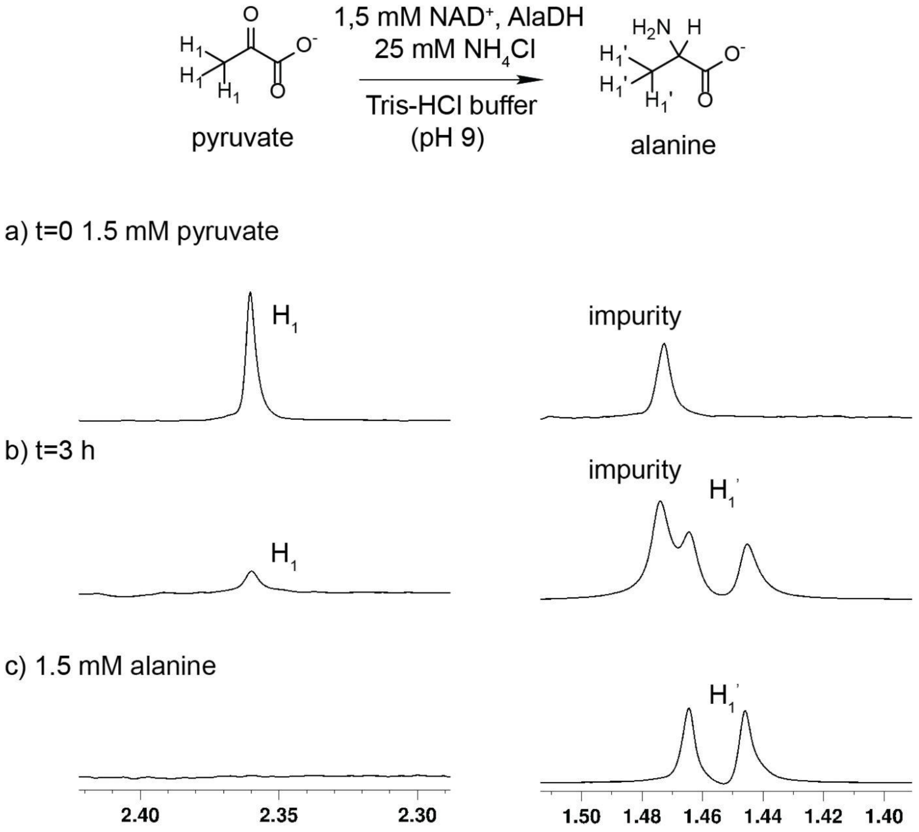

Supplementary Figure 13. ^{1}H NMR spectra of alanine formation from pyruvate. a) The initial spectrum. b) The spectrum after 3 h. c) The control alanine spectrum.

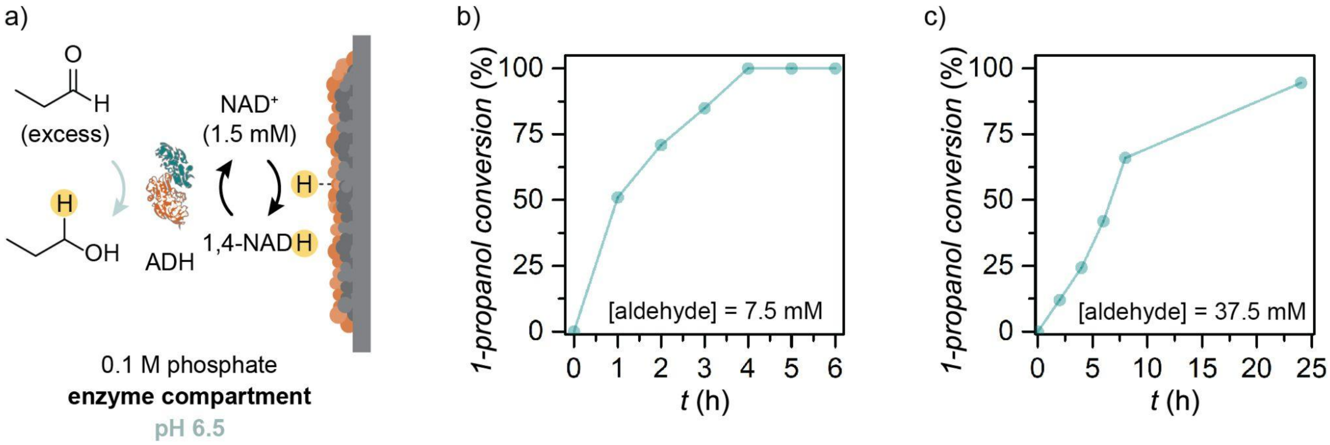

Supplementary Figure 14. Performance of aldehyde reduction in the BioPMR with excess aldehyde relative to NAD^+. a) Cell setup and idealized process. Reduction of b) 5-fold molar excess of propionic aldehyde with 10 units of ADH and c) 25-fold excess of propionic aldehyde with 30 units of ADH in phosphate buffer at pH 6.5. The reaction mixture contains 1 mg/mL BSA.

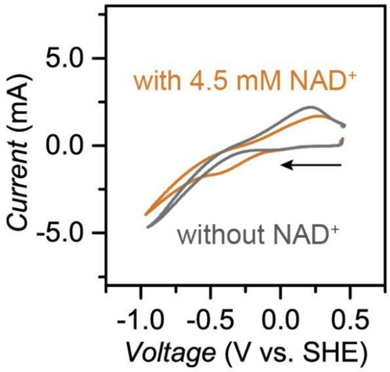

Supplementary Figure 15. CV measurements in tris buffer at pH 9 without NAD^+ (gray) and with 4.5 mM NAD^+ (orange). Taken with a Pt-coated Pd foil working electrode, a Ag/AgCl reference electrode, and a Pt mesh counter electrode. A scan rate of 250 mV/s was used and the cell was sparged with Ar prior to the measurement.

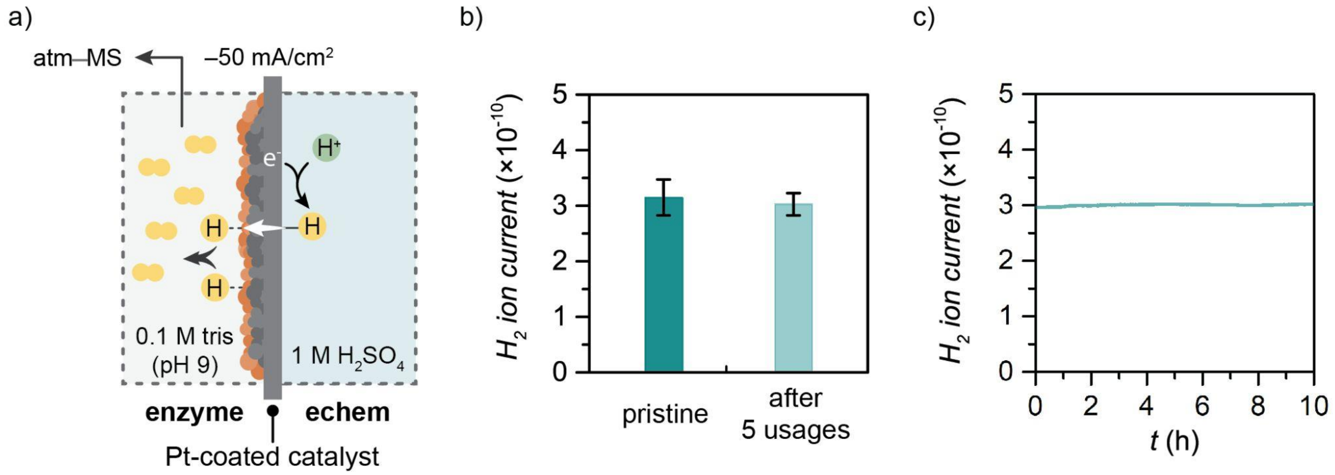

Supplementary Figure 16. Stability of H_2 flux with Pt-coated catalyst in tris buffer at pH 9 during electrolysis. a) Cell setup. b) H_2 gas evolution at steady-state as a function of foil usage for NADH regeneration. c) H_2 gas evolution at steady-state as a function of time after reaching steady-state. Error bars represent the standard deviation of triplicate measurements on different foils. The center point of each error bar represents the mean of the triplicate measurements.

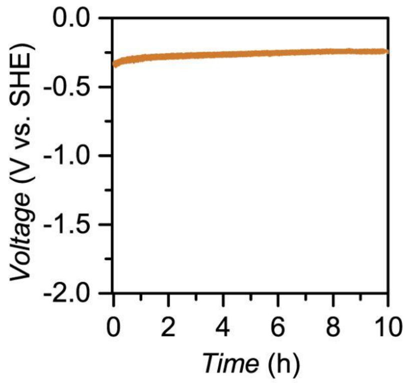

Supplementary Figure 17. The voltage range during electrolysis in the BioPMR. The electrolysis was performed at –50 mA·cm^{-2} over 10 h. The chemical compartment consisted of tris buffer at pH 9.
